# Supplementary material for: Involvement of a Multidrug Efflux Pump and Alterations in Cell Surface Structure in the Synergistic Antifungal Activity of Nagilactone E and Anethole against Budding Yeast Saccharomyces cerevisiae
Source: Antibiotics (Basel). 2021 May 6;10(5):537. doi: 10.3390/antibiotics10050537 (PMC8148520; doi:10.3390/antibiotics10050537)
Supplement: Supplementary file 1 [file antibiotics-10-00537-s001.zip › antibiotics-1203962-supplementary.pdf]

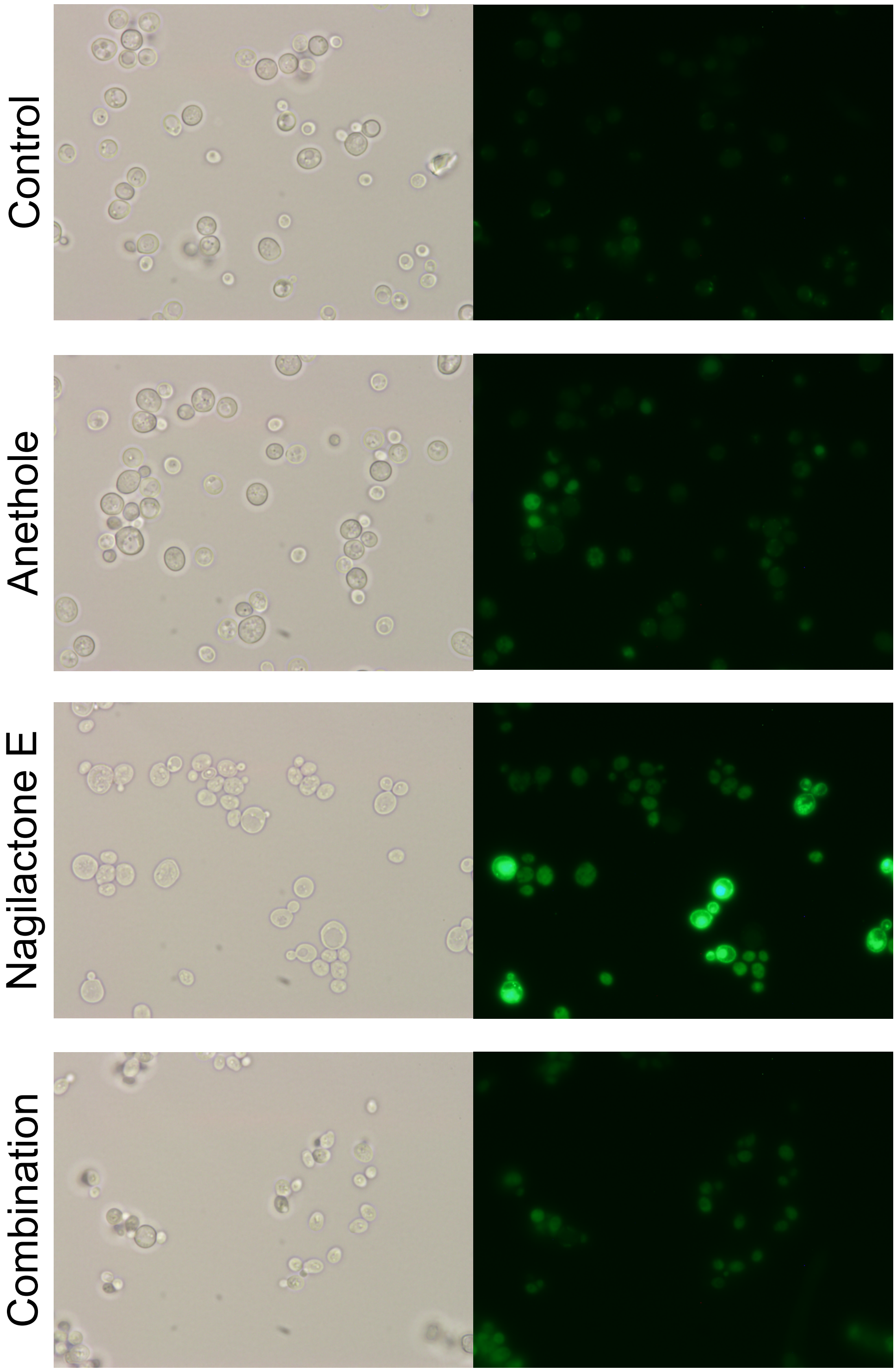

Supplementary Figure S1.
Phase-contrast and fluorescent micrographs of cells expressing Pdr5p tagged with green fluorescent protein. Exponentially growing cells were grown at 30˚C in 2.5% ME treated with 312.5 μM anethole, 250 μM nagilactone E, or their combination for 72 h. Control denotes treatment without drugs.

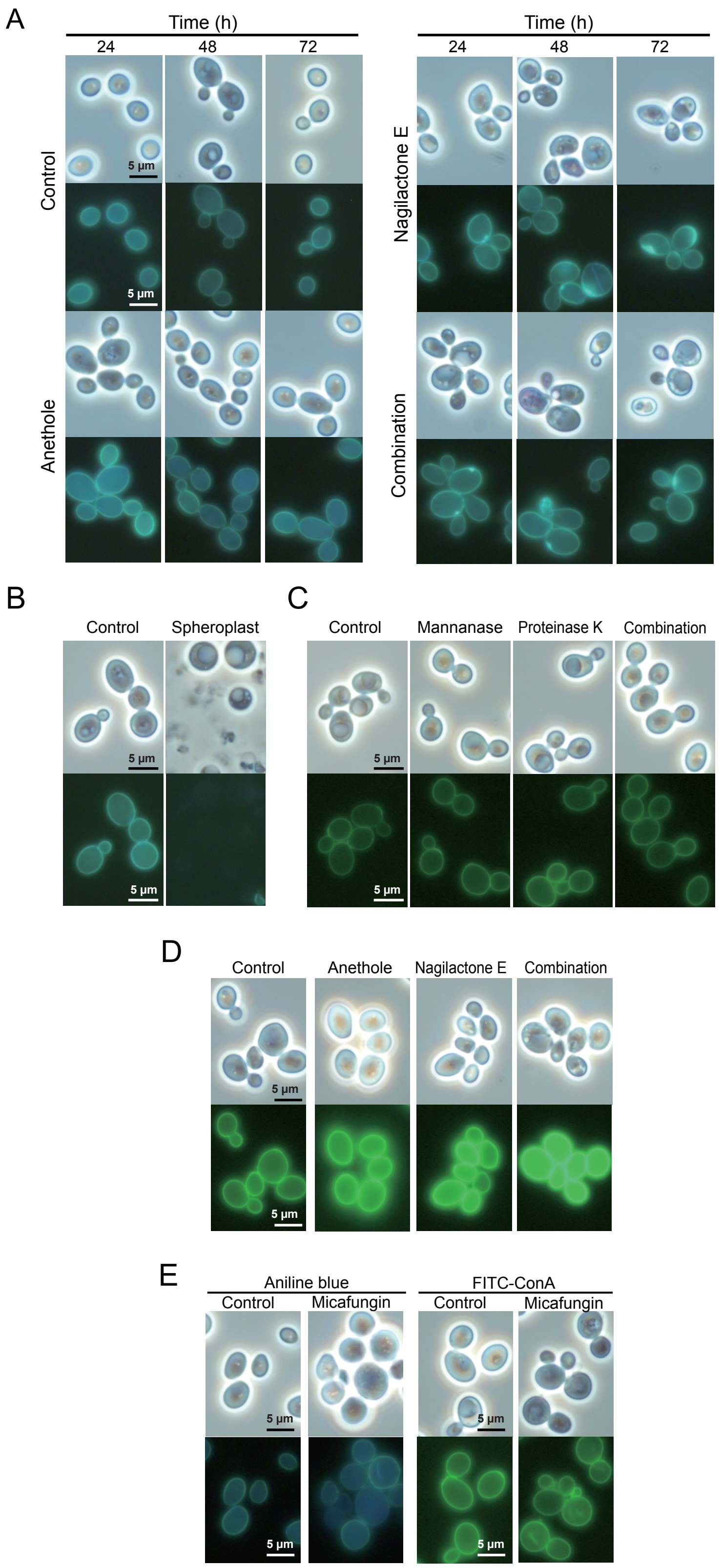

Supplementary Figure S2.

Visualization of β-glucan and mannan in yeast cells stained with aniline blue and FITC-ConA, respectively.

(A) Exponentially growing cells were grown at 30 ˚C in 2.5% ME treated with 312.5 μM anethole, 250 μM nagilactone E, or their combination. Control denotes treatment without drugs. A portion of cells were withdrawn at the indicated incubation time and then stained with aniline blue. (B) Exponentially growing cells were treated with or without zymolyase 20T at 30 ˚C for 30 min, and were then stained with aniline blue. (C) Exponentially growing cells were treated with mannanase and/or proteinase K. Control denotes treatment without enzymes. The cells were then stained with FITC-ConA. (D) Exponentially growing cells were grown at 30 ˚C for 72 h in 2.5% ME treated with 312.5 μM anethole, 250 μM nagilactone E, or their combination. Control denotes treatment without drugs, prior to staining with FITC-ConA. (E) Exponentially growing cells were grown at 30 ˚C for 72 h in 2.5% ME treated with 0.4 μM micafungin at 30 ˚C for 48 h, prior to staining with aniline blue or FITC-ConA.
